# Mean platelet volume may not differ in patients with idiopathic tinnitus^☆^

**DOI:** 10.1016/j.bjorl.2020.06.001

**Published:** 2020-07-18

**Authors:** Cengiz Beyan, Esin Beyan

**Affiliations:** a Ufuk University Faculty of Medicine, Ankara, Turkey; b University of Health Sciences, Kecioren Training and Research Hospital, Department of Internal Medicine, Ankara, Turkey

Dear Editor,

We read carefully the research of Yildiz et al. that was performed retrospectively in tinnitus patients,^1^ namely, that mean platelet volume (MPV) values were higher in patients with idiopathic tinnitus than the control group. We think that there are different factors that may have affected the MPV results in this study.

MPV measurement has not been standardized until today. It is not recommended being used for diagnosis or prognosis in acquired diseases since it has not yet been standardized.^2^ Following the collection of the blood to the blood tube, as soon as the platelets come into contact with ethylenediaminetetraacetic acid, which is used as an anticoagulant, their diameters increase up to 30% within the first two hours.^3^ Also, the difference of the devices used in the measurement is an important problem, and it has been reported that the measurement with different devices may cause up to 40% deviation in the MPV results.^4^ There was no description of the method in the study of Yildiz et al., and the fact that the time from blood collection to measurement as well as the devices used in the measurement were not specified caused the MPV results obtained unreliable. Moreover, since the study was carried out retrospectively, pre-analytical and analytical errors could not be excluded and it was reported that exclusion of analytical errors was very important, especially in MPV measurements.^5^ The age difference between the patient group and the control group also appears as a variable that negatively affected the MPV results.^2^ Finally, it was not clear how the normal MPV range used to classify MPV results as low, normal or high was determined. Essentially, the MPV obtained from healthy people should be determined according to normal values in the laboratory where the study was conducted, and since deviations occurred in MPV results with different devices, whichever instrument was studied with it must be specified.

As a result, MPV values may not differ in patients with idiopathic tinnitus.

## Conflicts of interest

The authors declare no conflicts of interest.
